# Subcritical Water as a Pre-Treatment of Mixed Microbial Biomass for the Extraction of Polyhydroxyalkanoates

**DOI:** 10.3390/bioengineering9070302

**Published:** 2022-07-08

**Authors:** Liane Meneses, Asiyah Esmail, Mariana Matos, Chantal Sevrin, Christian Grandfils, Susana Barreiros, Maria A. M. Reis, Filomena Freitas, Alexandre Paiva

**Affiliations:** 1 LAQV-REQUIMTE, Department of Chemistry, School of Science and Technology, NOVA University Lisbon, 2819-516 Caparica, Portugal; lp.meneses@campus.fct.unl.pt (L.M.); sfb@fct.unl.pt (S.B.); 2 UCIBIO—Applied Molecular Biosciences Unit, Department of Chemistry, School of Science and Technology, NOVA University Lisbon, 2819-516 Caparica, Portugal; a.esmail@campus.fct.unl.pt (A.E.); m.matos@campus.fct.unl.pt (M.M.); amr@fct.unl.pt (M.A.M.R.); 3 Associate Laboratory i4HB-Institute for Health and Bioeconomy, School of Science and Technology, NOVA University Lisbon, 2819-516 Caparica, Portugal; 4 CEIB-Interfaculty Research Centre of Biomaterials, University of Liege, B-4000 Liege, Belgium; csevrin@uliege.be (C.S.); c.grandfils@uliege.be (C.G.)

**Keywords:** polyhydroxyalkanoate (PHA), poly(3-hydroxybutyrate-co-3-hydroxyvalerate) (P(HB-co-HV)), mixed microbial culture (MMC), hypochlorite digestion, subcritical water (SBW)

## Abstract

Polyhydroxyalkanoate (PHA) recovery from microbial cells relies on either solvent extraction (usually using halogenated solvents) and/or digestion of the non-PHA cell mass (NPCM) by the action of chemicals (e.g., hypochlorite) that raise environmental and health hazards. A greener alternative for PHA recovery, subcritical water (SBW), was evaluated as a method for the dissolution of the NPCM of a mixed microbial culture (MMC) biomass. A temperature of 150 °C was found as a compromise to reach NPCM solubilization while mostly preventing the degradation of the biopolymer during the procedure. Such conditions yielded a polymer with a purity of 77%. PHA purity was further improved by combining the SBW treatment with hypochlorite digestion, in which a significantly lower hypochlorite concentration (0.1%, *v/v*) was sufficient to achieve an overall polymer purity of 80%. During the procedure, the biopolymer suffered some depolymerization, as evidenced by the lower molecular weight (M_w_) and higher polydispersity of the extracted samples. Although such changes in the biopolymer’s molecular mass distribution impact its mechanical properties, impairing its utilization in most conventional plastic uses, the obtained PHA can find use in several applications, for example as additives or for the preparation of graft or block co-polymers, in which low-M_w_ oligomers are sought.

## 1. Introduction

Over the past few decades, polyhydroxyalkanoate (PHA) production has drawn considerable attention and intensive work has been undertaken to convert these biodegradable and biocompatible polymers into viable competitors of oil-based plastics. Production processes have been optimized and their costs have already decreased significantly [1]. However, the downstream processes for polymer recovery from cells, and their purification, still hinder PHAs’ wider implementation [2,3]. Given the intracellular nature of PHAs, their release from the surrounding biomass requires cells to rupture, allowing the subsequent separation of the polymer from the non-PHA cell mass (NPCM) [4]. The procedures currently used consist of solvent extraction or digestion methods. Solvent extraction mainly requires halogenated solvents (e.g., chloroform, methylene chloride, 1,2-dichloroethane) for PHAs’ dissolution, which results in high extraction yields and highly pure biopolymers with low endotoxin contents [4,5,6]. Digestion methods, on the other hand, target the NPCM by the action of chemicals (e.g., sodium hypochlorite, acids), surfactants, enzymes, or biological agents, as reviewed by Pérez-Rivero et al. (2019) [6]. Although the digestion methods present a lower toxicity for human health and require a lower investment, they can affect the polymers’ properties while producing high volumes of wastewater which raise environmental concerns with recycling difficulties [6]. 

Due to its strong oxidizing properties, hypochlorite has been frequently used to degrade most of the NPCM (proteins, lipids, carbohydrates, nucleic acids) into water-soluble compounds. However, most of the studies reported in the literature are referring to a large range of this oxidation agent, making comparison very difficult between these investigations. However, in general, sodium hypochlorite was used at concentrations ranging from 1.05 wt.% to 12.1 wt.% [7,8,9,10,11], and large volumes of the reactant solution were required. Variable extraction yields (70–100 wt.%) and polymer purities (88–98%) were attained depending on the reaction time (1–12 h) and temperature (from room temperature to 37 °C) [7,8,9,10,11]. Although the procedure’s conditions can be adjusted to reach high extraction yields (>70 wt.%) and polymer purities (up to 98%), the treatment with hypochlorite affects the quality of the obtained PHA by impacting its molecular weight (M_w_) and polydispersity index (PDI) [2,3,12]. Hypochlorite digestion was reported to decrease the biopolymer’s M_w_ and to raise its PDI. Such alterations of PHAs’ quality may compromise the final polymer applications [5], particularly in terms of mechanical and thermal properties [11,13,14]. Indeed, PHAs with a M_w_ below 400 kDa have a lower resistance to mechanical stress [13,14], with a Mw above 600 kDa representing a threshold to fit to thermoplastic applications [11]. Another drawback of the procedure is the difficulty in completely removing traces of hypochlorite from the recovered PHA. Moreover, the utilization of hypochlorite can generate toxic halogenated compounds [4].

In line with this, considerable efforts have been made to develop alternative and greener methods for PHA recovery with high yields and purity degrees, comparable to those obtained with the use of organic solvents. Alternative solvents, such as ionic liquids [15] and supercritical CO_2_ [16], have been proposed but did not reach the desired PHA yield or purity degree. Due to its dissolution and hydrolysis capacity, subcritical water (SBW) has been used for the extraction of several added value compounds from different sources. At high pressure and temperature in the liquid state, the properties of water change: its density is slightly decreased when compared to water at an ambient pressure and temperature (around 900 mg/L), its dielectric constant also decreases with temperature, and its ionic product increases—reaching values as high as −12, while water at ambient conditions reaches −14 [17]. Such changes confer water with the ability to dissolve or even hydrolyze several macromolecules, including proteins, polysaccharides, and lipids [18]. SBW has been used for the extraction of phenolic compounds and carbohydrates from red and white wine grape pomace [19,20], as well as from cork [21]. It has also been used to obtain purified cellulose from sesame seed hulls [22]. Moreover, SBW has also demonstrated its capacity to extract proteins, peptides, and amino acids from codfish frames [23]. Therefore, due to its characteristics, SBW shows the potential to be used as a green solvent for PHA extraction, since it can hydrolyze and dissolve NPCM components such as the cell membrane’s proteins [23] and phospholipids [24].

This work focused on evaluating the feasibility of applying SBW for PHA recovery from the biomass of a mixed microbial culture (MMC). SBW treatment was applied at different temperatures for NPCM solubilization and the procedure’s efficiency was evaluated in terms of recovery yield and polymer purity, and through the polymer’s physical–chemical properties. Furthermore, the SBW treatment was also combined with hypochlorite digestion as a strategy to improve the extraction procedure. The obtained polymers were characterized for their composition, molecular mass distribution, thermal properties, and crystallinity. To the best of our knowledge, this work reports, for the first time, the use of SBW for PHA extraction in a manner that minimizes the utilization of harmful chemicals such as hypochlorite.

## 2. Materials and Methods

### 2.1. Biomass Production

The PHA-containing biomass was obtained by cultivation of an MMC in a three-stage bioprocess, as described in [25], using fermented fruit waste as a feedstock. To prevent PHA degradation, the cultivation broth collected from the final stage was acidified to pH 2–3 and stored at 4 °C until further use. Prior to the extraction experiments, the acidified cultivation broth was neutralized by the addition of NaOH 5 M and centrifuged (10,375× *g*, for 10 min, at 20 °C). The supernatant was discarded, while the biomass pellet was washed with deionized water and centrifuged again under the same conditions. The washed biomass was lyophilized for 48 h, at −50 °C and 0.8 mBar. The freeze-dried biomass was stored in closed plastic bags at −20 °C until required for the extraction experiments.

### 2.2. Polymer Extraction with Chloroform

Solvent extraction with chloroform was used as the reference extraction method, as well as to purify the PHA obtained from the different extraction methods, before the characterization processes. The polymer was extracted from the lyophilized biomass in a Soxhlet apparatus, as described in [26]. Briefly, 5 g of dried biomass was extracted with 250 mL chloroform, at 80 °C, over 24 h. The obtained polymer solution was left in a fume hood overnight for partial solvent evaporation. Afterwards, the polymer was precipitated in cold ethanol (1:10, *v/v*), under vigorous stirring. Finally, the polymer was dried at room temperature until reaching a constant weight.

### 2.3. NPCM Digestion with Sodium Hypochlorite

The lyophilized biomass (~0.2 g) was suspended in 5 mL of a sodium hypochlorite solution at a concentration of 5.0% (*v/v*). The digestion was conducted at room temperature, for 3 h as described in [9], under constant stirring (200 rpm). The samples were centrifuged (7012× *g*, 10 min, 20 °C) and the pellets were washed with deionized water until a neutral pH was reached. Finally, the polymer samples were freeze dried and kept in closed flasks, at room temperature.

### 2.4. Subcritical Water-Assisted Extraction

**Figure 1:**
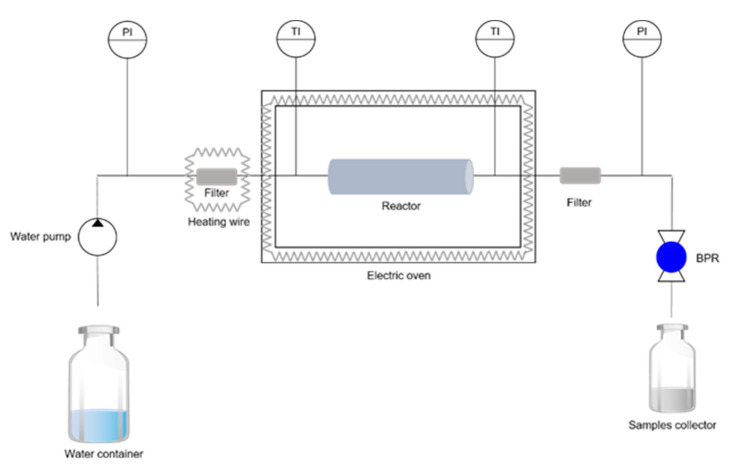
Schematic representation of the SBW apparatus used for SBW treatment. PI: pressure indicators; TI: temperature indicators.

The lyophilized biomass was subjected to SBW treatment at different temperatures between 130 and 200 °C, using an SBW apparatus (Figure 1) operated as described in [20]. Briefly, the HiP stainless-steel reactor was filled with lyophilized biomass between two layers of glass spheres (6 mm diameter) and placed inside the electric oven. Water was pumped into the reactor through a high-pressure tube at a flow rate of 10 mL/min, using a Knauer 40 preparative pump 1800 coupled to a Rheonik Rhe 01.03 unit. After pressurizing the system, the heating cords and the oven were turned on. The water temperature increased at an average rate of 1.2 °C/min until the desired value was reached and maintained for 30 min. The pressure of the entire system was maintained by a Tescom back pressure regulator (BPR), set at 80 bar. The recovered biomass was collected from the reactor and dried in an oven at 60 °C, until it reached a constant weight. The polymer content in the samples was determined by Soxhlet extraction with chloroform, as described in Section 2.1.

### 2.5. Hypochlorite Digestion of the SBW Treated Biomass

Dried SBW-treated (150 °C) biomass samples (~0.2 g) were suspended in deionized water (5 mL) and digested with different hypochlorite contents, namely, 0.1, 0.5, 3.0, and 5.0% (*v/v*). The digestion was conducted as described in Section 2.3. The polymer samples were freeze dried and kept in closed flasks, at room temperature.

### 2.6. Calculations

For each procedure, the recovery yield (%) was determined as follows:

(1) $$Recovery\;yield=\frac{m_{product}}{m_{biomass}}\times 100\;\%$$

where *m_product_* is the dry mass of the sample (g) obtained with each extraction procedure and *m_biomass_* is the mass of the dry biomass (g) used for each procedure. In the case of the standard extraction with chloroform, the recovery yield corresponds to the extracted PHA. For the SBW treatments and hypochlorite extraction, the recovery yield corresponds to a mixture of PHA and NPCM. The purity of the samples was determined by gas chromatography (GC) as described in [26], with slight modifications. Briefly, samples (2–5 mg) were hydrolyzed with 1 mL 20% (*v/v*) sulfuric acid in methanol solution and 1 mL heptadecane in chloroform (1 g/L), at 100 °C for 3.5 h. After hydrolysis, 1 mL of deionized water was added. After separation of the organic and aqueous phases, the organic phase, with the resulting methyl esters, was analyzed in a chromatograph 430-GC Bruker equipped with a Crossbond, Stabilwax column (Restek, Bellefonte, PA, USA). Analysis was performed at a constant 14.5 psi pressure, using helium as a carrier gas. The calibration curve was prepared with poly(3-hydroxybutyrate-co-3-hydrovalerate), P(HB-co-HV), (Sigma-Aldrich, Saint Louis, MA, USA, 88 mol% 3-HB, 12 mol% 3-HV) dissolved in chloroform at concentrations ranging from 0.053 to 6.750 g/L, with heptadecane as an internal standard (1.0 g/L).

### 2.7. Polymer Characterization

All polymer samples used for characterization procedures were previously purified from the remaining NPCM, using Soxhlet extraction with chloroform as described in Section 2.1.

#### 2.7.1. Composition

The polymers’ compositions were determined by GC as described in Section 2.6. 

#### 2.7.2. Molecular Mass Distribution

The polymers’ molecular weight (M_w_ and M_n_) and polydispersity index (PDI) were analyzed by size exclusion chromatography (SEC). For this analysis, 15 mg polymer samples was dissolved in 15 mL of chloroform for 18 h at room temperature. The samples were then filtered with glass fiber filters (47 mm, PALL). The analysis was performed in a Waters Millennium system with chloroform as an eluent with a rate of 1 mL/min. Relative average molecular weights were determined against polystyrene standards using the universal calibration curve.

#### 2.7.3. Thermal Properties

Differential scanning calorimetry (DSC) analysis was performed using a differential scanning calorimeter DSC 131 (Setaram, Caluire, France). The samples were placed in aluminum crucibles and analyzed in a temperature range between −90 and 220 °C, with heating and cooling speeds of 10 °C/min. Thermogravimetric analysis (TGA) was performed using the thermogravimetric equipment Labsys EVO (Setaram, Caluire, France). Samples were placed in aluminum crucibles and analyzed in a temperature range between 25 and 500 °C, at 10 °C/min. The crystallinity index (X_c_, %) of the samples was estimated as the ratio between the melting enthalpy (ΔHm, J g^−1^) of its melting peak and the melting enthalpy of 100% crystalline P(3HB), previously reported to be 146 J g^−1^ [27].

## 3. Results and Discussion

### 3.1. Solvent Extraction with Chloroform and Hypochlorite Digestion

The original biomass had a PHA content of 66 wt.%, which was determined by Soxhlet extraction with chloroform. The obtained polymer was a 3-hydroxybutyrate and 3-hydroxyvalerate (3-HB/3-HV) co-polymer, P(HB-co-HV), with a 3-HV content of 18 wt.% (Figure S1), which is within the range reported for P(HB-co-HV) produced by MMC from several agri-food wastes (13–24 wt.%) [25,28,29,30].

Soxhlet extraction with chloroform, of the original biomass, was performed as a reference method for the comparison of the original polymer’s properties with those obtained with hypochlorite digestion and SBW treatments. The procedure resulted in an extraction yield of 66 wt.% and a purity of 91 wt.% (Table 1). The chloroform-extracted polymer had a M_w_ of 3.0 × 10^5^ g/mol and a low PDI of 1.3 (Table 1), which shows its homogeneity in terms of molecular chain length. These values are of the same order of magnitude as those reported for P(HB-co-HV) obtained from MMC by extraction with chloroform (M_w_ between 2.5 × 10^5^ and 6.4 × 10^5^ g/mol; PDI between 1.3 and 2.5) [25,28,29,30]. The polymer’s thermal properties, melting temperature (T_m_) of 153 °C and degradation temperature (T_deg_) of 283 °C (Table 1), are also within the values reported for other P(HB-co-HV) samples extracted from MMC biomass, namely, a T_m_ between 147 and 168 °C and T_deg_ between 247 and 278 °C [28,29]. The polymer presented an X_c_ of 34%, which is in agreement with the values reported for other P(HB-co-HV) samples (18–69%) [31,32,33].

Hypochlorite digestion of the MMC biomass resulted in a higher recovery yield (75 wt.%) compared to the chloroform extraction procedure (66 wt.%), but the sample’s purity was considerably lower (77 wt.%, compared to 91 wt.% for the chloroform-extracted sample) (Table 1), showing that the solubilization of NPCM by hypochlorite was not complete. Nevertheless, this procedure took only 3 h at room temperature, while the Soxhlet extraction with chloroform took 24 h at a temperature of 80 °C (Table 1). Moreover, simpler equipment is used, and sodium hypochlorite is not as hazardous as chloroform, with a lower environmental impact. The polymer’s M_w_ (3.2 × 10^5^ g/mol) and its PDI (1.3) were in the same order of magnitude as those reported for P(HB-co-HV) recovered from biomass by hypochlorite digestion (3.6 × 10^5^–6.3 × 10^5^ g/mol and 1.7, respectively) [34,35]. These data show there was no impact on the polymer’s molecular mass distribution compared to the chloroform-extracted sample; however, a decrease in the sample’s T_m_ to 140 °C (Table 1) was observed, as well as a reduction in crystallinity from 34% to 21% (Table 1).

### 3.2. SBW-Assisted Extraction

#### 3.2.1. SBW Treatment

**Figure 2:**
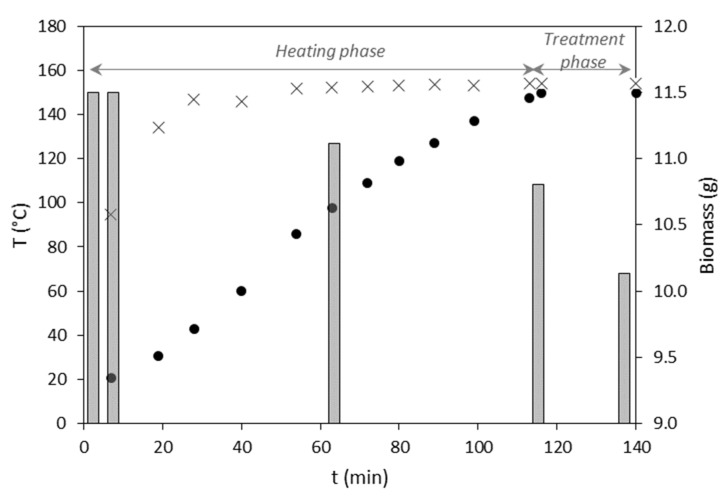
Heating profile of the SBW system (temperature of the water entering (×) and exiting (●) the reactor) and mass of the sample (bars) in the reactor during the assay performed at 150 °C.

SBW treatment was applied to the MMC biomass samples at temperatures in the range of 130–200 °C. An example of the SBW process is shown in Figure 2. Briefly, the water flow was initiated at room temperature (18–20 °C), and, after being pressurized, the system was heated until the water exiting the reactor attained the desired temperature, which was maintained for 30 min. Approximately 2 h of heating was required for the water exiting the reactor to achieve the desired temperature (in this case, 150 °C), although at the inlet it was reached within less than 1 h. Within the nearly 2 h heating phase of the experiment, there was a mass loss of 0.7 g from the initial 11.5 g of biomass placed inside the reactor, while during the 30 min treatment phase at 150 °C, a further 0.65 g was removed. This corresponds to an overall mass loss of 12%.

As shown in Table 1, by increasing the temperature of the SBW treatment from 130 to 150 °C, there was a decrease in the recovery yield from 94 wt.% to 88 wt.%, accompanied by an increase in the sample’s purity from 67 wt.% to 77 wt.%. This indicates that the mass lost during the SBW treatment resulted mostly from the solubilization/hydrolysis of NPCM. However, a reduction in the polymer’s M_w_, concomitant with an increase in its PDI, was observed for both samples compared to the chloroform and the hypochlorite-extracted samples. This impact was more pronounced for the SBW treatment at 150 °C, in which the obtained polymer had a M_w_ of 5.0 × 10^4^ g/mol and a PDI of 6.0 (Table 1). These results indicate that the PHA molecules were depolymerized to some extent during the SBW treatment. Moreover, this depolymerization appeared to be correlated with the applied temperature, being accentuated when raising the extraction temperature.

The thermal degradation of PHA occurs mainly by random chain scission of the ester bonds, resulting in a gradual reduction in M_w_ by the formation of smaller polymer fractions, including short oligomers and monomers [36,37]. Studies on P(3HB) show that at temperatures between 160 and 180 °C, there is a significant decrease in the polymer’s thermal stability which becomes drastic at temperatures above 200 °C [37,38]. Regarding the co-polymer P(HB-co-HV), which has a higher thermal stability than the homopolymer, a significant decrease in residual mass was only observed at 230 °C [39]. Even though these authors did not observe significant mass alterations, the analysis of M_w_ and PDI showed that, when the polymer was subjected to 180 °C for 30 min, the M_w_ decreased by 80%. Increasing the temperature caused even larger decreases in M_w_ [39]. Hence, exceeding the polymer’s melting point seems to be the trigger to initiate chain scissions [39] and, with the exception of SBW treatment at 130 °C, all the temperatures applied in this study were close to, or above, the T_m_ of P(HB-co-HV). Moreover, the combination of multiple factors can lead to an accelerated degradation process. In this work, we combined temperature and pressure in the SBW treatment, which can explain the reduction in M_w_ and increase in PDI, even at lower temperatures than those found in the literature.

Increasing the temperature of the SBW treatment to 165 and 180 °C resulted in lower recovery yields (77 and 58 wt.%, respectively) and a lower polymer purity (75 and 66 wt.%, respectively), which suggests that the thermal depolymerization led to the release of soluble monomers and/or short oligomers, which were removed from the sample. This is supported by the observation that the sample extracted at 180 °C had the lowest M_w_ (7.0 × 10^3^ g/mol), but the PDI was also lower (3.9) (Table 1), suggesting that the short oligomers generated during the procedure were probably solubilized in SBW and washed away.

Further increasing the SBW temperature to 200 °C led to the destruction of the sample and no polymer was recovered after SBW treatment. These results agree with the reported stability of P(HB-co-HV) co-polymers [38,39].

**Figure 3:**
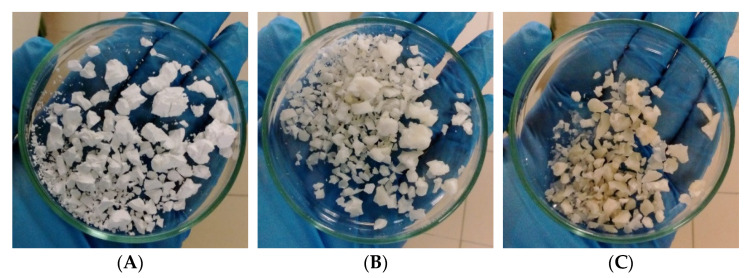
Images of the samples obtained after the SBW treatment of MMC biomass at (**A**) 150 °C, (**B**) 165 °C, and (**C**) 180 °C.

These changes in polymers’ molecular chains impacted their thermal properties, with a gradual decrease in the T_m_ when raising the temperature during the SBW treatment. As highlighted in Table 1, the polymers recovered from the MMC biomass subjected to 165 °C and 180 °C had their T_m_ decreased by almost 10 °C and over 20 °C, respectively. However, between 130 and 150 °C, the T_m_ decreased by only 3 °C. These changes in the thermal characteristics of the polyesters were also noticed in terms of crystallinity percentage. The SBW treatment of 130 °C displayed X_c_ values (35%) closer to that of P(HB-co-HV) extracted with chloroform (34%). In contrast, materials submitted to SBW treatments at higher temperatures presented higher X_c_ values, increasing from 6% for 150 °C, to 14% for 165 °C, and 17% for 180 °C (Table 1). Figure 3 shows the P(HB-co-HV) obtained at the temperatures that caused an increase in X_c_, and the changes to its visual aspect are also noticeable: the PHA becomes harder and glossier. Such phenomena have been reported for other biopolymers extracted by SBW, e.g., cellulose [22]. Interestingly, no significant changes were observed for the samples regarding the T_deg_ of these polymers, which ranged between 287 and 294 °C, whatever the experimental conditions of purification.

The PHA’s thermal properties, especially T_m_, are related to other properties, namely, M_w_, the presence of side chains, or the presence of other functional groups [40]. A direct relation between T_m_ and M_w_, has been proposed by some authors [41], and it has been reported that for low-M_w_ PHA (10^3^–10^4^ g/mol), the correspondent T_m_ values are within 146–153 °C [42], corroborating the relation between the decrease in T_m_ with the decrease in M_w_.

Combining the highest recovery yield (88 wt.%) and polymer purity (77 wt.%), the SBW treatment at 150 °C was selected for subsequent tests. Although there was a reduction in the polymer’s M_w_ and an increase in its PDI, the thermal properties remained practically unchanged, compared to the chloroform-extracted PHA.

#### 3.2.2. Extraction of PHA from the SBW-Treated Biomass with Hypochlorite

Being demonstrated to be effective, SBW treatment was employed at 150 °C as a pre-treatment of the MMC biomass prior to digestion with sodium hypochlorite.

Hypochlorite concentrations ranging from 0.1 to 5.0% (*v/v*) were used to determine the most appropriate conditions to improve the polymer’s recovery yield and purity compared to the SBW treatment alone. As shown in Table 1, a higher polymer purity (84 wt.%) was achieved for the SBW-treated samples subjected to the highest hypochlorite concentration (5.0% *v/v*). For the other concentrations tested, 3.0, 0.5, and 0.1% (*v/v*), higher recovery yields were obtained (83–85 wt.%), with a reduction in the polymer’s purity (80–82 wt.%). Even though the purity values achieved with the combination of SBW and hypochlorite digestion are not as high as those obtained with chloroform extraction (91 wt.%), they are still higher than those achieved with hypochlorite digestion alone (77 wt.%).

This two-step purification technique had no impact on the P(HB-co-HV) composition, with all polymers having the same 3HV content for the sample obtained with the SBW treatment at 150 °C (17 wt.%) (Table 1). On the other hand, the polymers’ crystallinity index values were noticed to rise with the combined treatment method (48–62%, Table 1) in parallel with only using SBW. This can be linked to the greater purity of the extracted polymers. Regarding the M_w_, it remained the same for all tested conditions (2.8 × 10^4^–3.0 × 10^4^ g/mol, respectively) and was only slightly lower than the 150 °C SBW-treated sample (5.0 × 10^4^ g/mol). However, even though the M_w_ decreased, with the exception of the sample digested with 0.1% hypochlorite, the extracted polymer became more homogeneous, as evidenced by the decrease in the PDI, from 6 (after SBW 150 °C) to 3.1–4.8, for 5.0, 3.0, and 0.5% (*v/v*) hypochlorite. These changes could be assigned to the higher extraction of the lower M_w_ PHA oligomers formed during the SBW treatment upon treating with hypochlorite. The sample digested with the lowest hypochlorite concentration (0.1%, *v/v*) was characterized by having a higher PDI (7.5), indicating that these conditions yielded a polymer with a wide variety of chain sizes. The lower hypochlorite concentration might not have been enough to solubilize and wash away the smaller PHA oligomers that were thus retained within the recovered sample.

The impact of the different hypochlorite concentrations tested might be related to the alkalinity afforded by this oxidant compound. Berger et al. (1989) previously reported that when using a hypochlorite solution with pH 10, the obtained polymer had a M_w_ 2.5 times higher than when using the hypochlorite solution without pH correction (13.6) [7]. The pH of the hypochlorite solutions used was 9.1, 8.6, 7.3, and 6.7 for 5, 3, 0.5, and 0.1% (*v/v*) hypochlorite, respectively.

As has been previously discussed, these changes in the size of the polymeric chains are also evidenced by the alterations in the thermal properties of the P(HB-co-HV). There was a decrease in the polymers’ T_m_ by 6–11 °C compared to the polymer obtained directly after SBW at 150 °C, and 12–17 °C when compared to the original polymer (Table 1). These changes might be related to alterations on the samples’ M_w_ and PDI [38,41,42,43]. The polymers’ thermal degradation was apparently not affected, with the T_deg_ still in the range of 276–290 °C.

### 3.3. Overall Assessment of the SBW-Hypochlorite Procedure

These findings show that, after being subjected to the SBW treatment at 150 °C, a hypochlorite concentration 50 times lower can be used to reach a higher PHA recovery yield and a higher polymer purity, compared to hypochlorite digestion alone. Examples in the literature show that using only hypochlorite extraction, some authors were able to achieve purities of 90% (using 1% NaOCl, for 3 h, at room temperature) [34]. Others [10] were even able to scale up the extraction to 50 L, maintaining a purity of 93%; however, this method employed a higher hypochlorite concentration (13% NaOCl), for 1 h at room temperature. To achieve polymers with even higher purity degrees, hypochlorite can be combined with organic solvents (chloroform or acetone), or even surfactants (sodium dodecyl sulfate or triton X-100).

SBW hydrolysis alone was not a suitable method for PHA extraction, since the low temperatures tested (130 °C) had a low solubilization capacity, while the higher temperatures (180–200 °C) caused severe polymer degradation. Intermediate temperatures, on the other hand, caused some polymer degradation, as shown by the lower M_w_ and higher PDI of the extracted samples. Coupling the SBW treatment with hypochlorite digestion allowed a reduction of the amount of hypochlorite required to reach a good extraction yield and polymer purity, which renders this an approach of interest.

The observed decrease in the PHA’s M_w_ impairs the utilization of the biopolymer in most of the conventional applications of plastics due to the loss of mechanical properties. Nevertheless, there are specific applications for which low-M_w_ PHAs are of interest, such as their utilization as plasticizers [44] and surfactants, or for the preparation of graft and block co-polymers, which are useful for the fabrication of bio-adhesives, drug-delivery systems, drug-coating systems, and tissue-engineering materials [45,46,47]. Nevertheless, for biomedical and pharmaceutical uses, the biopolymers would require further purification to meet the requirements of such high-value areas. For such applications, the PHA molecules are depolymerized into monomers or short oligomers (10^2^–10^4^ g/mol) by physical (e.g., thermal decomposition), chemical methods (e.g., acid hydrolysis, alcoholysis), and/or enzymatic methods [46,47,48]. The developed SBW–hypochlorite digestion methodology could be of great value for the extraction of the biopolymer with the appropriate M_w_ for use in such areas of application, without the need to resort to the chemical hydrolysis of high-M_w_ PHA.

## 4. Conclusions

To minimize the impact of harmful chemicals in the purification process of PHA, the use of SBW was validated in this work. It was observed that a two-step purification process integrating SBW pre-treatment at 150 °C, which showed the best compromise between NPCM solubilization and PHA properties, and hypochlorite digestion allowed the dissolvement of the rest of the NPCM material. This approach showed that, after the SBW treatment, it was possible to recover the PHA with 50 times less hypochlorite, with a purity higher than that obtained with hypochlorite alone. Moreover, the impact on the PHA’s M_w_ was mostly due to the SBW treatment, since after the hypochlorite extraction, the difference was less significant. Reducing the utilization of chemicals, such as hypochlorite, is of the utmost importance due to their environmental burden. The low-M_w_ PHA generated can find use as additives, surfactants, and graft or block co-polymers, among other uses. With this work, it was shown that it is possible to develop methods for the extraction of PHA using green solvents, with a lower environmental impact than the conventional solvents used.

## Figures and Tables

**Table 1:** Thermal properties and molecular mass distribution of the samples obtained with the different extraction methods tested (T_m_, melting temperature; T_deg_, degradation temperature; M_w_, average molecular weight; PDI, polydispersity index; X_c_, crystallinity index; 3HV, 3-hydroxyvalerate; RT, room temperature).

Extraction Method	Recovery Yield (%)	Polymer Purity (%)	3-HV Content (wt.%)	M_w_ (g/mol)	PDI	T_m_ (°C)	T_deg_ (°C)	X_c_ (%)
Chloroform (Soxhlet, 80 °C, 24 h)	66 ± 0.92	91 ± 0.82	18 ± 1.1	3.0 × 10^5^	1.3	153	283	34
Hypochlorite (5.0%, RT, 3 h)	75 ± 1.1	77 ± 0.91	17 ± 0.10	3.2 × 10^5^	1.3	140	277	21
SBW-assisted extraction								
*SBW treatment*								
130 °C	94 ± 1.3	67 ± 0.67	18 ± 1.1	1.2 × 10^5^	5.1	150	278	35
150 °C	88 ± 1.4	77 ± 0.77	17 ± 0.10	5.0 × 10^4^	6.0	147	295	40
165 °C	77 ± 1.4	75 ± 0.79	16 ± 0.91	2.3 × 10^4^	7.4	141	294	48
180 °C	58 ± 1.0	66 ± 1.06	15 ± 1.3	7.0 × 10^3^	3.9	127	291	51
200 °C	2	(*)	(*)	(*)	(*)	(*)	(*)	(*)
*SBW treatment (150 °C) + Hypochlorite*								
0.1%	85 ± 1.4	80 ± 0.80	17 ± 1.1	3.0 × 10^4^	7.5	136	290	62
0.5%	83 ± 1.3	81 ± 0.59	17 ± 1.1	3.0 × 10^4^	4.8	139	287	48
3.0%	83 ± 1.2	82 ± 0.67	17 ± 1.2	3.0 × 10^4^	3.1	140	282	49
5.0%	81 ± 1.3	84 ± 0.69	17 ± 1.1	2.8 × 10^4^	3.9	141	276	59

(*) The sample was degraded.

## Data Availability

The data presented in this study are available on request from the corresponding authors.

## Supplementary Materials

The following supporting information can be downloaded at: https://www.mdpi.com/article/10.3390/bioengineering9070302/s1, Figure S1: GC chromatogram of the P(HB-co-HV) sample obtained by Sohxlet extraction with chloroform, to illustrate the typical profile obtained.

## References

[1] Kourmentza C., Plácido J., Venetsaneas N., Burniol-Figols A., Varrone C., Gavala H.N., Reis M.A.M. (2017). Recent Advances and Challenges towards Sustainable Polyhydroxyalkanoate (PHA) Production. Bioengineering.

[2] Mannina G., Presti D., Montiel-Jarillo G., Suárez-Ojeda M.E. (2019). Bioplastic Recovery from Wastewater: A New Protocol for Polyhydroxyalkanoates (PHA) Extraction from Mixed Microbial Cultures. Bioresour. Technol..

[3] Samorì C., Abbondanzi F., Galletti P., Giorgini L., Mazzocchetti L., Torri C., Tagliavini E. (2015). Extraction of Polyhydroxyalkanoates from Mixed Microbial Cultures: Impact on Polymer Quality and Recovery. Bioresour. Technol..

[4] Koller M., Niebelschütz H., Braunegg G. (2013). Strategies for Recovery and Purification of Poly [(R)-3-Hydroxyalkanoates] (PHA) Biopolyesters from Surrounding Biomass. Eng. Life Sci..

[5] Pagliano G., Galletti P., Samorì C., Zaghini A., Torri C. (2021). Recovery of Polyhydroxyalkanoates from Single and Mixed Microbial Cultures: A Review. Front. Bioeng. Biotechnol..

[6] Pérez-Rivero C., López-Gómez J.P., Roy I. (2019). A Sustainable Approach for the Downstream Processing of Bacterial Polyhydroxyalkanoates: State-of-the-Art and Latest Developments. Biochem. Eng. J..

[7] Berger E., Ramsay B.A., Ramsay J.A., Chavarie C. (1989). PHA Recovery by Hypochlorite Digestion of Non-PHB Biomass. Biotechnol. Tech..

[8] López-Abelairas M., García-Torreiro M., Lú-Chau T., Lema J.M., Steinbüchel A. (2015). Comparison of Several Methods for the Separation of Poly(3-Hydroxybutyrate) from Cupriavidus necator H16 Cultures. Biochem. Eng. J..

[9] Villano M., Valentino F., Barbetta A., Martino L., Scandola M., Majone M. (2014). Polyhydroxyalkanoates Production with Mixed Microbial Cultures: From Culture Selection to Polymer Recovery in a High-Rate Continuous Process. New Biotechnol..

[10] Heinrich D., Madkour M.H., Al-Ghamdi M.A., Shabbaj I.I., Steinbüchel A. (2012). Large Scale Extraction of Poly (3-Hydroxybutyrate) from Ralstonia eutropha H16 Using Sodium Hypochlorite. AMB Express.

[11] Lorini L., Martinelli A., Pavan P., Majone M., Valentino F. (2021). Downstream Processing and Characterization of Polyhydroxyalkanoates (PHAs) Produced by Mixed Microbial Culture (MMC) and Organic Urban Waste as Substrate. Biomass Convers. Biorefinery.

[12] Kunasundari B., Sudesh K. (2011). Isolation and Recovery of Microbial Polyhydroxyalkanoates. Express Polym. Lett..

[13] Guzik M., Witko T., Steinbüchel A., Wojnarowska M., Sołtysik M., Wawak S. (2020). What Has Been Trending in the Research of Polyhydroxyalkanoates? A Systematic Review. Front. Bioeng. Biotechnol..

[14] Serafim L.S., Queirós D., Rossetti S., Lemos P.C., Koller M. (2016). Biopolymer Production by Mixed Microbial Cultures: Integrating Remediation with Valorization. Recent Advances in Biotechnology Microbial Biopolyester, Vol. 1: Production, Performance and Processing Microbiology, Feedstocks, and Metabolism.

[15] Kobayashi D., Fujita K., Nakamura N., Ohno H. (2015). A Simple Recovery Process for Biodegradable Plastics Accumulated in Cyanobacteria Treated with Ionic Liquids. Appl. Microbiol. Biotechnol..

[16] Hejazi P., Vasheghani-farahani E., Yamini Y. (2003). Supercritical Fluid Disruption of Ralstonia eutropha for Poly (-Hydroxybutyrate) Recovery. Biotechnol. Prog..

[17] Kruse A., Dinjus E. (2007). Hot Compressed Water as Reaction Medium and Reactant. Properties and Synthesis Reactions. J. Supercrit. Fluids.

[18] Brunner G. (2009). Near Critical and Supercritical Water. Part I. Hydrolytic and Hydrothermal Processes. J. Supercrit. Fluids.

[19] Pedras B., Salema-Oom M., Sá-Nogueira I., Simões P., Paiva A., Barreiros S. (2017). Valorization of White Wine Grape Pomace through Application of Subcritical Water: Analysis of Extraction, Hydrolysis, and Biological Activity of the Extracts Obtained. J. Supercrit. Fluids.

[20] Pedras B.M., Regalin G., Sá-Nogueira I., Simões P., Paiva A., Barreiros S. (2020). Fractionation of Red Wine Grape Pomace by Subcritical Water Extraction/Hydrolysis. J. Supercrit. Fluids.

[21] Cunha M., Lourenço A., Barreiros S., Paiva A., Simões P. (2020). Valorization of Cork Using Subcritical Water. Molecules.

[22] Zhang R.Y., Liu H.M., Hou J., Yao Y.G., Ma Y.X., Wang X. (2021). De Cellulose Fibers Extracted from Sesame Hull Using Subcritical Water as a Pretreatment. Arab. J. Chem..

[23] Melgosa R., Marques M., Paiva A., Bernardo A., Fernández N., Sá-Nogueira I., Simões P. (2021). Subcritical Water Extraction and Hydrolysis of Cod (Gadus Mmorhua) Frames to Produce Bioactive Protein Extracts. Foods.

[24] Tran Nguyen P.L., Go A.W., Huynh L.H., Ju Y.H. (2013). A Study on the Mechanism of Subcritical Water Treatment to Maximize Extractable Cellular Lipids. Biomass Bioenergy.

[25] Matos M., Cruz R.A.P., Cardoso P., Silva F., Freitas E.B., Carvalho G., Reis M.A.M. (2021). Combined Strategies to Boost Polyhydroxyalkanoate Production from Fruit Waste in a Three-Stage Pilot Plant. ACS Sustain. Chem. Eng..

[26] Cruz M.V., Araújo D., Alves V.D., Freitas F., Reis M.A.M. (2016). Characterization of Medium Chain Length Polyhydroxyalkanoate Produced from Olive Oil Deodorizer Distillate. Int. J. Biol. Macromol..

[27] Morais C., Freitas F., Cruz M.V., Paiva A., Dionísio M., Reis M.A.M. (2014). Conversion of Fat-Containing Waste from the Margarine Manufacturing Process into Bacterial Polyhydroxyalkanoates. Int. J. Biol. Macromol..

[28] Albuquerque M.G.E., Martino V., Pollet E., Avérous L., Reis M.A.M. (2011). Mixed Culture Polyhydroxyalkanoate (PHA) Production from Volatile Fatty Acid (VFA)-Rich Streams: Effect of Substrate Composition and Feeding Regime on PHA Productivity, Composition and Properties. J. Biotechnol..

[29] Duque A.F., Oliveira C.S.S., Carmo I.T.D., Gouveia A.R., Pardelha F., Ramos A.M., Reis M.A.M. (2014). Response of a Three-Stage Process for PHA Production by Mixed Microbial Cultures to Feedstock Shift: Impact on Polymer Composition. New Biotechnol..

[30] Matos M., Cruz R.A.P., Cardoso P., Silva F., Freitas E.B., Carvalho G., Reis M.A.M. (2021). Sludge Retention Time Impacts on Polyhydroxyalkanoate Productivity in Uncoupled Storage/Growth Processes. Sci. Total Environ..

[31] Sankhla I.S., Bhati R., Singh A.K., Mallick N. (2010). Poly(3-Hydroxybutyrate-Co-3-Hydroxyvalerate) Co-Polymer Production from a Local Isolate, Brevibacillus invocatus MTCC 9039. Bioresour. Technol..

[32] Bossu J., Angellier-Coussy H., Totee C., Matos M., Reis M., Guillard V. (2020). Effect of the Molecular Structure of Poly (3-Hydroxybutyrate-Co-3-Hydroxyvalerate) (P (3HB-3HV)) Produced from Mixed Bacterial Cultures on Its Crystallization and Mechanical Properties. Biomacromolecules.

[33] Esmail A., Pereira J.R., Sevrin C., Grandfils C., Menda U.D., Fortunato E., Oliva A., Freitas F. (2021). Preparation and Characterization of Porous Scaffolds Based on Poly (3-Hydroxybutyrate) and Poly (3-Hydroxybutyrate-Co-3-Hydroxyvalerate). Life.

[34] Martínez-Abad A., Cabedo L., Oliveira C.S.S., Hilliou L., Reis M.A.M., Lagarón J.M. (2016). Characterization of Polyhydroxyalkanoate Blends Incorporating Unpurified Biosustainably Produced Poly (3-Hydroxybutyrate-Co-3- Hydroxyvalerate). J. Appl. Polym. Sci..

[35] Pradhan S., Dikshit P.K., Moholkar V.S., Katiyar V., Kumar A., Mulchandani N. (2020). Production, Characterization, and Applications of Biodegradable Polymer: Polyhydroxyalkanoates. Advances in Sustainable Polymers. Materials Horizons: From Nature to Nanomaterials.

[36] Naser A.Z., Deia I., Darras B.M. (2021). Poly (lactic acid) (PLA) and polyhydroxyalkanoates (PHAs), green alternatives to petroleum-based plastics: A review. RSC Adv..

[37] Lorini L., Martinelli A., Capuani G., Frison N., Reis M., Ferreira B.S., Villano M., Majone M., Valentino F. (2021). Characterization of Polyhydroxyalkanoates Produced at Pilot Scale from Different Organic Wastes. Front. Bioeng. Biotechnol..

[38] Wang S., Chen W., Xiang H., Yang J., Zhou Z., Zhu M. (2016). Modification and Potential Application of Short-Chain-Length Polyhydroxyalkanoate (SCL-PHA). Polymers.

[39] Xiang H., Wen X., Miu X., Li Y., Zhou Z., Zhu M. (2016). Thermal Depolymerization Mechanisms of Poly(3-Hydroxybutyrate-Co-3-Hydroxyvalerate). Prog. Nat. Sci. Mater. Int..

[40] Palmieri S., Tittarelli F., Sabbatini S., Cespi M., Bonacucina G., Eusebi A.L., Fatone F., Stipa P. (2021). Effects of Different Pre-Treatments on the Properties of Polyhydroxyalkanoates Extracted from Sidestreams of a Municipal Wastewater Treatment Plant. Sci. Total Environ..

[41] Stanley A., Murthy P.S.K., Vijayendra S.V.N. (2020). Characterization of Polyhydroxyalkanoate Produced by Halomonas venusta KT832796. J. Polym. Environ..

[42] Cha S.H., Son J.H., Jamal Y., Zafar M., Park H.S. (2016). Characterization of Polyhydroxyalkanoates Extracted from Wastewater Sludge under Different Environmental Conditions. Biochem. Eng. J..

[43] Martínez-Herrera R.E., Alemán-Huerta M.E., Almaguer-Cantú V., Rosas-Flores W., Martínez-Gómez V.J., Quintero-Zapata I., Rivera G., Rutiaga-Quiñones O.M. (2020). Efficient Recovery of Thermostable Polyhydroxybutyrate (PHB) by a Rapid and Solvent-Free Extraction Protocol Assisted by Ultrasound. Int. J. Biol. Macromol..

[44] Hong S.G., Hsu H.W., Ye M.T. (2013). Thermal Properties and Applications of Low Molecular Weight Polyhydroxybutyrate. J. Therm. Anal. Calorim..

[45] Yu G.-E. (2008). Process of Producing Low Molecular Weight Poly (Hydroxyalkanoate)s from High Molecular Weight Poly (Hydroxyalkanoate)s.

[46] Chaber P., Kwiecień M., Zięba M., Sobota M., Adamus G. (2017). The heterogeneous selective reduction of PHB as a useful method for preparation of oligodiols and surface modification. RSC Adv..

[47] Kanmani P., Kumaresan K., Aravind J., Karthikeyan S., Balan R. (2016). Enzymatic degradation of polyhydroxyalkanoate using lipase from Bacillus subtilis. Int. J. Environ. Sci. Technol..

[48] Don T.-M., Liao K.-H. (2018). Studies on the alcoholysis of poly(3-hydroxybutyrate) and the synthesis of PHB-b-PLA block copolymer for the preparation of PLA/PHB-b-PLA blends. J. Polym. Res..

