# Supplementary material for: Subcritical Water as a Pre-Treatment of Mixed Microbial Biomass for the Extraction of Polyhydroxyalkanoates
Source: Bioengineering (Basel). 2022 Jul 8;9(7):302. doi: 10.3390/bioengineering9070302 (PMC9311994; doi:10.3390/bioengineering9070302)
Supplement: Supplementary file 1 [file bioengineering-09-00302-s001.zip › bioengineering-1774529-supplementary.pdf]

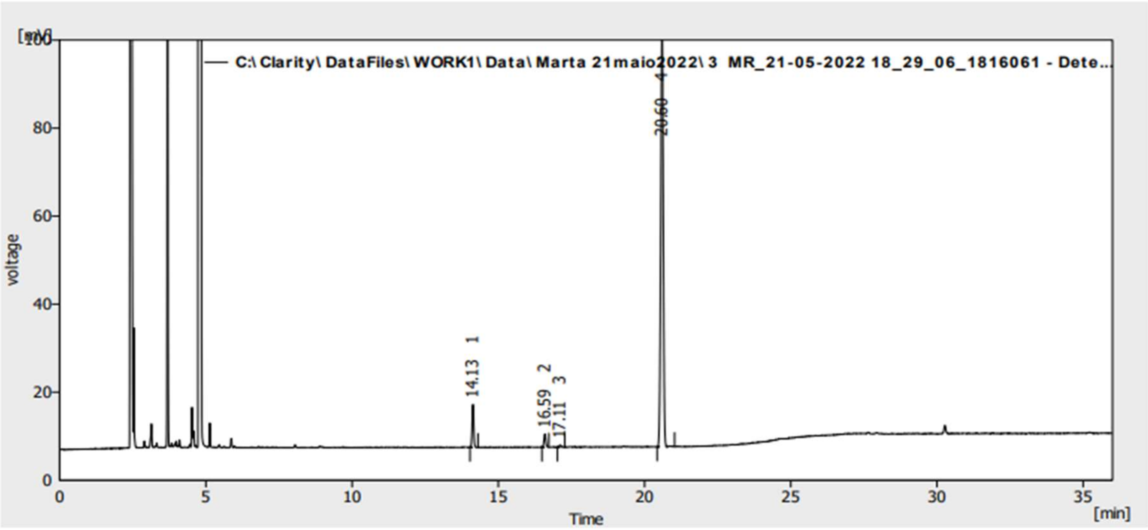

Result Table (Uncal - C:\Clarity\DataFiles\WORK1\Data\Marta 21maio2022\3 MR_21-05-2022 18_29_06_1816061 - Detector 1)

	Reten. Time [min]	Area [mV.s]	Height [mV]	Area [%]	Height [%]	W05 [min]	Compound Name
1	14.130	38.998	9.739	6.3	8.9	0.07	
2	16.593	13.183	3.008	2.1	2.8	0.07	
3	17.113	2.297	0.480	0.4	0.4	0.07	
4	20.598	564.153	95.961	91.2	87.9	0.09	
	Total	618.632	109.188	100.0	100.0		

Figure S1: GC chromatogram of the P(HB-co-HV) sample obtained by Sohxlet extraction with chloroform, to illustrate the typical profile obtained.
